# Optimized Anchor-Modified Peptides Targeting Mutated RAS Are Promising Candidates for Immunotherapy

**DOI:** 10.3389/fimmu.2022.902709

**Published:** 2022-05-26

**Authors:** Renato B. Baleeiro, Louisa S. Chard Dunmall, Peng Liu, Shuangshuang Lu, Yuchun Lone, Nicholas R. Lemoine, Yaohe Wang

**Affiliations:** ^1^ Centre for Cancer Biomarkers and Biotherapeutics, Barts Cancer Institute, Queen Mary University of London, London, United Kingdom; ^2^ Sino-British Research Centre for Molecular Oncology, National Centre for International Research in Cell and Gene Therapy, School of Basic Medical Sciences, Academy of Medical Sciences, Zhengzhou University, Zhengzhou, China; ^3^ Centre National de la Recherche Scientifique (CNRS), Transgénèse et Archivage d’Animaux Modèles (TAAM), Orleans, France

**Keywords:** RAS, neo-antigens, cancer immunotherapy, cancer vaccine, cancer peptides, T-cells

## Abstract

RAS mutations occur in approximately 20% of all cancers and given their clonality, key role as driver mutation, association with poor prognosis and undruggability, they represent attractive targets for immunotherapy. We have identified immunogenic peptides derived from codon 12 mutant RAS (G12A, G12C, G12D, G12R, G12S and G12V), which bind to HLA-A*02:01 and HLA-A*03:01 and elicit strong peptide-specific CD8+ T cell responses, indicating that there is an effective CD8+ T-cell repertoire against these mutant RAS-derived peptides that can be mobilized. Alterations in anchor residues of these peptides enhanced their binding affinity to HLA-A*02:01 molecules and allowed generation of CD8+ T cells that responded to target cells pulsed with the anchor-modified and also with the original peptide. Cytotoxic T cells generated against these peptides specifically lysed tumor cells expressing mutant RAS. Vaccination of transgenic humanized HLA-A2/DR1 mice with a long peptide encompassing an anchor-modified 9-mer G12V epitope generated CD8+ T cells reactive to the original 9-mer and to a HLA-A*02:01-positive human cancer cell line harboring the G12V mutation. Our data provide strong evidence that mutant RAS can be targeted by immunotherapy.

## Introduction

Cancer immunotherapy is one of the most promising and rapidly advancing cancer treatment modalities (1–4). The promise of immunotherapy is particularly evident in the recent success of antibodies targeting cytotoxic T-lymphocyte antigen 4 (CTLA-4) and programmed cell death protein 1 (PD1)/PD-L1 immune checkpoints in promoting anti-tumor immune responses and extending survival in a number of cancer types (4). Although antibodies targeting CTLA-4 and PD-1/PD-L1 represent a major breakthrough in cancer immunotherapy, application of checkpoint inhibitors in certain tumors have not led to significant tumor regression. This may be due to lack of pre-existing immune response to the tumor antigens.

One approach to promoting this anti-tumor immune response is cancer vaccination, which triggers specific immune responses to tumor antigens and can promote development of long-term anti-tumor memory (5). Historically, cancer vaccines have targeted tumor-associated antigens (TAAs), which are self-antigens whose expression is limited to certain tissues but are found ectopically expressed or overexpressed in cancer cells. Unfortunately, most clinical trials targeting TAAs have failed to demonstrate durable benefit compared with standard of care treatment (6).

Recently, genomic sequencing and bioinformatics platforms have enabled rapid identification of neo-antigens expressed by individual patients that are potentially more clinically tractable vaccine targets (7). Neo-antigens are tumor-specific antigens resulting from somatic DNA alterations and have emerged as the ideal tumor antigens. They are tumor specific and “non-self” in the sense that they are not subjected to negative thymic selection and, therefore, potentially immunogenic. However, because the vast majority are unique to each patient, they require the application of personalized therapies. Moreover, most mutations are non-driver mutations and may be expressed only by a proportion of the population of tumor cells (8).

The proteins HRAS, KRAS and NRAS are the products of proto-oncogenes that appear mutated in approximately 20% of human cancers (9). Single point mutation at codon 12, 13, or 61 causes aberrant RAS function, leading to the uncontrolled cellular proliferation associated with cancer (10), which places RAS as a key driver mutation. Mutation at these conserved sites favors GTP binding and promotes constitutive activation of RAS. All three RAS isoforms share sequence identity in all of the regions that are responsible for GDP/GTP binding, GTPase activity, and effector interactions.

Activating RAS mutations are present in up to 95% of pancreatic ductal adenocarcinoma (PDAC) patients (11–13), with the G12D substitution being the most frequent (49.5%), followed by G12V (30%), G12R (12%), G12C (3%), G12S (2.5%) and G12A (2%) (14). In lung cancer it appears mutated in about 35% of patients and the most common mutations are G12C (44.2%), G12V (21.4%) and G12D (19.7%), followed by G12A (7.3%), G12S (5%) and G12R (2.5%) (14). Approximately 50% of colorectal carcinoma patients have RAS mutations, with the most common being G12D (43.6%), followed by G12V (26.2%), G12C (12.3%), G12S (8.9%), G12A (7.7%) and G12R (1.2%) (14). The frequency and tumor-specificity of RAS mutations make them attractive therapeutic targets, but efforts to inhibit RAS and its downstream effectors have so far shown no clinical success, suggesting RAS as an inherently undruggable target (15) - with the exception of G12C, which is the target of a promising inhibitory drug (16). A more attractive approach therefore may be to use RAS neo-antigens as vaccine or T cell therapy targets. This approach is supported by a number of observations, including the identification of spontaneous mutant KRAS-specific T cells in PDAC patients (17); demonstrable efficacy of re-infused *ex vivo* expanded spontaneous CD8+ T cells recognizing KRAS G12D in an HLA-C*08:02 colorectal cancer patient (18); and autologous T cell transfer protocols using murine T cell receptors (TCRs) directed against HLA-A*11:01-restricted RAS G12D that could delay tumor growth in immune-deficient mouse xenograft models (19). Peptide vaccination approaches against mutant RAS are sparsely reported, but collectively demonstrate effective induction of mutant RAS T-cell immune responses, albeit with no significant impact on survival in clinical studies (20–22).

The limited clinical success in inducing long-lasting anti-tumor efficacy suggests a significant opportunity for improvement of mutant RAS targeting strategies. Here, we use a bioinformatics approach (23, 24) to predict HLA-binding peptides from mutant RAS followed by cell-based binding assays (25) and T cell assays to analyze immunogenicity against such potential antigenic peptides. Because CD8+ T cell responses might be difficult to generate against low affinity peptides (26, 27), we also tested amino acid substitutions within the anchor position in the peptide sequence to increase HLA-binding affinity. We demonstrate that human CD8+ T cells can be readily generated against peptides derived from the six most common RAS mutations G12A, G12C, G12D, G12R, G12S and G12V in the context of HLA-A*02:01 and HLA-A*03:01. A particular alteration in anchor residue of a G12V 9-mer peptide enhanced its binding affinity to HLA-A*02:01 molecules, rendering this peptide more immunogenic and leading to the generation of CD8+ T cells that responded to target cells pulsed with the anchor-modified and with the original peptide. Furthermore, cytotoxic T cells generated against this peptide specifically lysed tumor cells expressing mutant RAS. As a proof-of-principle of this approach, immunization of transgenic humanized mice expressing HLA-A*02:01 with a long peptide containing the anchor-modified residue generated CD8+ T cells that recognize the original 9-mer peptide and a human HLA-A*02:01-positive pancreatic cancer cell line bearing the G12V mutation. These data demonstrate that CD8+ T cells capable of reacting against this 9-mer mutant RAS peptide are present in the repertoire and can be mobilized by an anchor-modified peptide, providing strong evidence that this anchor-modified peptide can be used in immunotherapy protocols to treat RAS-positive malignancies.

## Results

### Selection of HLA-A*02:01, HLA-A*03:01 and HLA-A*11:01-Binding Mutated RAS Epitopes

**Figure 1:**
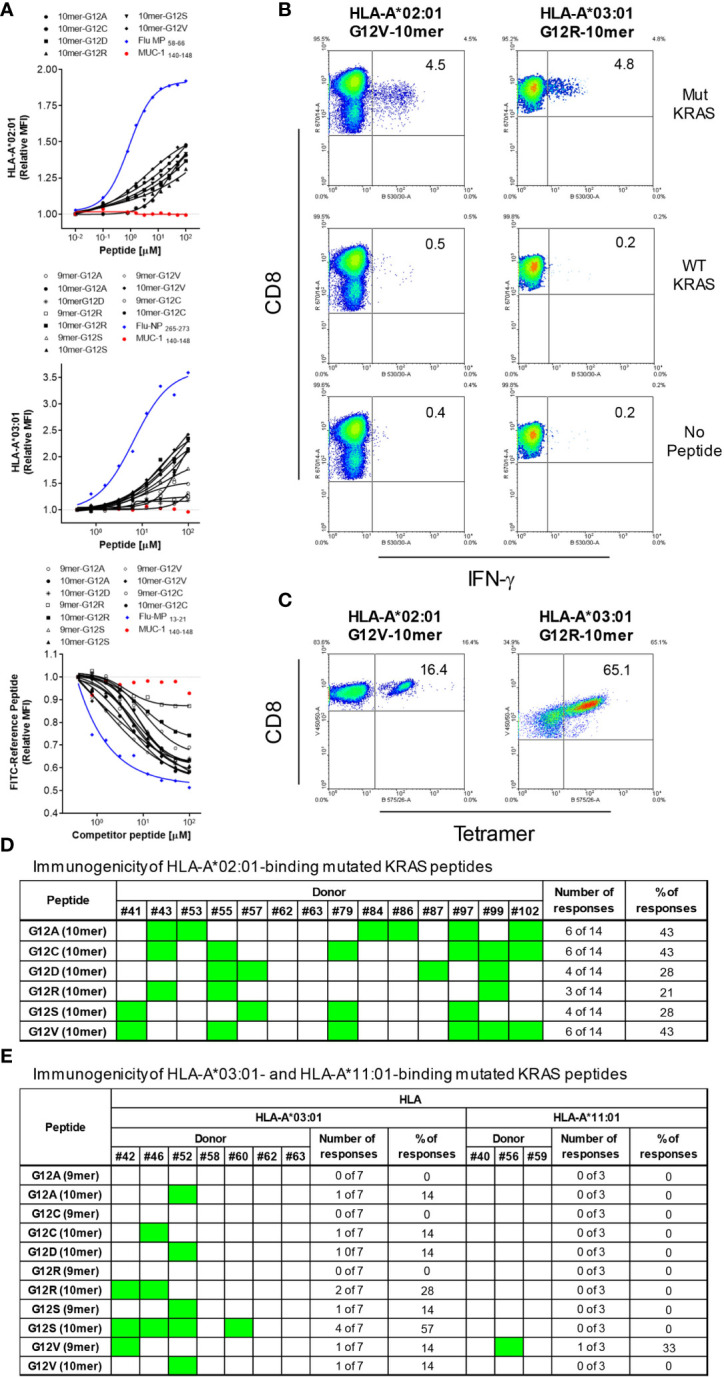
HLA-binding and immunogenicity of RAS-mutant peptides. MHC-I Stabilization assays using TAP-deficient T2 cell lines expressing HLA-A*02:01 **(A,** upper graph**)** or HLA-A*03:01 **(A,** middle graph**)** or a competitive MHC-I-binding assay using a lymphoblastoid cell line (CJO-A) expressing HLA-A*11:01 **(A,** lower graph**)** were carried out to assess binding of 9-and 10-mer peptides representing 6 RAS mutants. The binding of each peptide was analyzed three times and the mean of all of the experiments performed is shown. MUC-1 140-148 peptide was used as negative control; as positive control, Flu MP 58-66 and Flu-NP 265-273 peptides were used for HLA-A*02:01 and HLA-A*03:01 respectively; Flu-MP 13-21 peptide was used as positive control for HLA-A*11:01; Flu-MP: Influenza virus Matrix Protein; Flu-NP: Influenza virus Nucleoprotein. To assess immunogenicity, CD8+ T cells from healthy donors were stimulated four times with autologous peptide-pulsed mature dendritic cells. Seven days after the last round of stimulation, CD8+ T cells were challenged with T2 cells pulsed with the same RAS mutants, WT counterpart or left without peptide. Intracellular IFN-γ was measured by FACS. The dot plots in **(B)** depict the RAS mutations that elicited CD8+ T cell response to a 10-mer peptide containing the G12V and G12R mutation from one representative donor expressing HLA-A*02:01 and HLA-A*03:01, respectively. CD8+ T cells from some responders were further stimulated and expanded and stained with tetramers. A representative experiment is show in **(C)**. The response from all 14 HLA-A*02:01-positive donors tested is shown in **(D)**. In **(E)** is shown the responses from 7 and 3 HLA-A*03:01- and HLA-A*11:01-positive donors, respectively; responses are shown in green. It is noteworthy that the CD8+ T cells reactive to the mutant RAS did not respond to the non-mutated RAS.

Using the NetMHC 4.0 program that predicts HLA-peptide binding (28), we identified RAS-derived epitopes that can bind to HLA-A*02:01, HLA-A*03:01 and HLA-A*11:01. Peptides that harbor the six most common mutations of RAS amino acid 12 were predicted to bind to these three HLA-A alleles. While only 10-mers were predicted to bind to HLA-A*02:01, 9-mers and 10-mers were predicted to bind to HLA-A*03:01 and HLA-A*11:01 with reasonably good affinities (**Supplementary Table 1**). The binding capacity of these peptides was further confirmed through cell-based binding assays. Although all peptides predicted to bind to the HLA-A alleles were revealed as true binders in the binding assays, there were some differences between predicted and actual binding capacity. The 10-mer G12A was predicted to be the best HLA-A*02:01 binder followed by the G12V and G12C, but the cell-binding assay revealed G12V as the best binder followed by G12A and G12S. Overall, 9-mers were predicted by NetMHC 4.0 to be better binders than the 10-mers to both HLA-A*03:01 and HLA-A*11:01. However, with the exception of the G12V HLA-A*11:01, the 10-mers outperformed the 9-mers in the binding assays (**Figure 1A** and **Supplementary Table 2**).

### Immunogenicity of Mutated RAS Epitopes

RAS peptides were assessed for their capacity to elicit CD8+ T cell responses. *In vitro* differentiated mature DCs loaded with each individual peptide were used to stimulate autologous CD8+ T cells in four rounds of stimulation. Seven days after the last round of stimulation, CD8+ T cells were challenged with T2 cells or monocytes pulsed with the mutant RAS epitope, its wild type counterpart or left without peptide and IFN-γ production used as a measure of epitope-specific T cell response. All six RAS 10-mer epitopes induced specific CD8+ T cells in the context of HLA-A*02:01 (**Figures 1B–D**). Interestingly, the immunogenicity varied among the donors, with a given donor responding to up to four mutations, but never to all of them (**Figures 1D, E**). The G12A, G12C and G12V were the most immunogenic epitopes, inducing specific T cells in 43% of the HLA-A*02:01-positive donors tested. G12D and G12S triggered specific T cells in 28% of the donors, while G12R did so in 21% of HLA-A*02:01-positive donors. The G12S and G12R 10-mer epitopes were the most immunogenic among the HLA-A*03:01-binding epitopes, eliciting specific CD8+ T cells in 57% and 28% of the donors, respectively. G12A, G12C, G12D and G12V were less immunogenic, showing reactivity in only 14% of the HLA-A*03:01-positive donors. None of the HLA-A*03:01-positive donors responded to the 9-mers G12A, G12C, G12R and G12S (**Figure 1E**). Interestingly, among the HLA-A*11:01-binding peptides, only the 9-mer G12V peptide was able to trigger a specific CD8+ T cell response in a HLA-A*11:01 donor (**Figure 1E**). Importantly, the specificity of the response against the mutated RAS peptides was very high, as in all cases the CD8+ T cells reactive to the mutant RAS peptide did not recognize cells pulsed with wild type RAS.

### Modification of Anchor Residues Enhances the Binding of RAS Peptides to HLA-A*02:01

**Figure 2:**
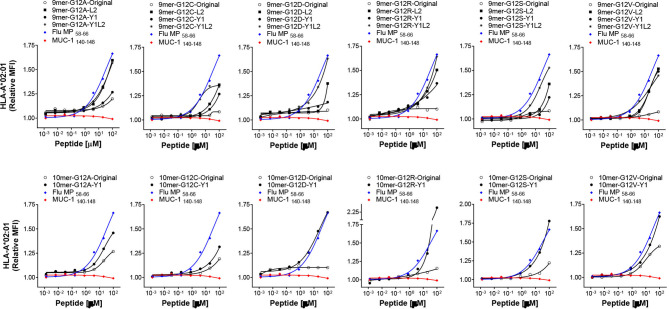
HLA-binding of anchor-modified RAS-mutant peptides. MHC-I Stabilization assays using TAP-deficient T2 cell lines expressing HLA-A*02:01 were carried out to assess binding of 9-and 10-mer anchor-modified peptides representing 6 RAS mutants. The binding of each peptide was analyzed three times and the mean of all of the experiments performed is shown. MUC-1 140-148 and Flu-MP 58-66 peptides were used as negative and positive control, respectively.

Although RAS-derived epitopes were able to bind to HLA-A*02:01 and HLA-A*03:01 and trigger specific CD8+ T cells, their immunogenicity varied greatly depending on the mutation and the donor. To try to improve immunogenicity of RAS epitopes we developed an approach to enhance affinity and, as a consequence, immunogenicity of poorly immunogenic RAS epitopes with low/moderate affinity for HLA-A*02:01. Alterations comprised of the modification in peptide sequence by replacing the anchor amino acids at position 1 with tyrosine (Y1) (for 9-mers and 10-mers), position 2 with leucine (L2) (for 9-mers) and position 1 and 2 with a tyrosine and leucine (Y1L2) (for 9-mers). All modifications were predicted to enhance affinity of the peptides for the HLA-A*02:01 (**Table 1**). To confirm the improvement in binding capacity, we tested all peptides in cell-based binding assays. All anchor-modified HLA-A*02:01-binding epitopes showed enhanced binding to the HLA molecule (**Figure 2** and **Table 1**).

**Table 1:** Predicted vs actual binding capacity of mutant original and anchor-modified RAS peptides.

Mutation	Peptide lenght	Peptide type	aa Sequence	Aff (nM) NetMHC	EC50 (µM) Binding assay
**G12A**	9mer	Original	LVVVGA**A**GV	1520	191.1
		Anchor-modified	**Y**VVVGA**A**GV	164.1	116.7
			L**L**VVGA**A**GV	91	17.54
			**YL**VVGA**A**GV	14.3	16.41
	10mer	Original	KLVVVGA**A**GV	237.8	113.9
		Anchor-modified	**YL**VVVGA**A**GV	65.6	27.74
**G12C**	9mer	Original	LVVVGA**C**GV	2088.8	619
		Anchor-modified	**Y**VVVGA**C**GV	250.8	137.6
			L**L**VVGA**C**GV	141.5	73.46
			**YL**VVGA**C**GV	18.5	26.09
	10mer	Original	KLVVVGA**C**GV	373.6	220.1
		Anchor-modified	**Y**LVVVGA**C**GV	95.7	95.49
**G12D**	9mer	Original	LVVVGA**D**GV	4317.5	453
		Anchor-modified	**Y**VVVGA**D**GV	565.9	199.3
			L**L**VVGA**D**GV	260.5	83.38
			**YL**VVGA**D**GV	28.4	13.42
	10mer	Original	KLVVVGA**D**GV	498	422.5
		Anchor-modified	**Y**LVVVGA**D**GV	140.5	5.683
**G12R**	9mer	Original	LVVVGA**R**GV	8610.8	429
		Anchor-modified	**Y**VVVGA**R**GV	2001.1	51.62
			L**L**VVGA**R**GV	971.3	23.7
			**YL**VVGA**R**GV	102.6	15.6
	10mer	Original	KLVVVGA**R**GV	506.9	271.8
		Anchor-modified	**L**LVVVGA**R**GV	150.1	6.55
**G12S**	9mer	Original	LVVVGA**S**GV	3297.9	580.5
		Anchor-modified	**Y**VVVGA**S**GV	364.3	206.7
			L**L**VVGA**S**GV	174	90.57
			**YL**VVGA**S**GV	20.6	21.17
	10mer	Original	KLVVVGA**S**GV	390.7	185.1
		Anchor-modified	**Y**LVVVGA**S**GV	109	10.65
**G12V**	9mer	Original	LVVVGA**V**GV	1710.3	610
		Anchor-modified	**Y**VVVGA**V**GV	196.3	28.26
			L**L**VVGA**V**GV	124	25.56
			**YL**VVGA**V**GV	17	22.95
	10mer	Original	KLVVVGA**V**GV	300.2	68.14
		Anchor-modified	**Y**LVVVGA**V**GV	79.2	15.47

The actual binding capacity of the peptides to HLA-A*02:01 was subsequently assessed in a T2 cell binding assay and EC50 values in μM for each peptide are shown. Anchor-modified amino acids appear in red. The mutant amino acid in each peptide appear in bold.

Binding affinity of original and anchor-modified mutant RAS peptides was predicted by NetMHC 4.0 program and affinity in nM is shown.

### Immunogenicity of Anchor-Modified G12V RAS Peptides

**Figure 3:**
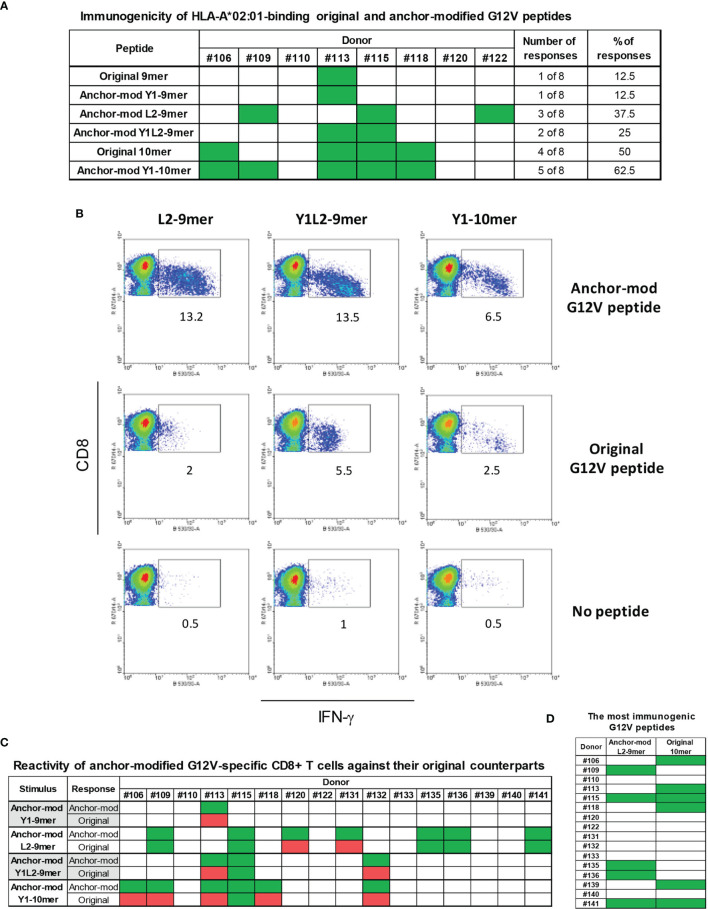
Immunogenicity of anchor-modified G12V RAS-mutant peptides. To assess immunogenicity of anchor-modified G12V RAS peptides, CD8+ T cells from healthy donors were stimulated four times with autologous peptide-pulsed mature dendritic cells. Seven days after the last round of stimulation, CD8+ T cells were challenged with T2 cells pulsed with the same RAS mutants (anchor-modified or original), original or left without peptide. Intracellular IFN-γ was measured by FACS. The response from all 8 HLA-A*02:01-positive donors tested so far is shown in **(A)**. The dot plots in **(B)** depict the RAS mutations that elicited CD8+ T cell response to three anchor-modified G12V peptides from a representative donor expressing HLA-A*02:01; and the response from all 16 donors tested **(C)**; responses are shown in green - in cases where T cells stimulated against an anchor-modified peptide did not respond to its original counterpart are marked in red. In **(D)** is shown all donors who were stimulated with anchor-modified 9-mer L2 that recognized the original 9-mer and original 10-mer.

We next sought to ascertain whether the modifications in the anchor residues of 9-mer and 10-mer peptides render them more immunogenic without altering their antigenic specificity. As a proof-of-concept for this approach, we chose the G12V mutation, the second most common RAS alteration across all tumors (14). To that end, *in vitro* differentiated DCs from eight HLA-A*02:01-positive donors were loaded with individual G12V-RAS peptides (Original 9-mer and 10-mer, or anchor-modified Y1-9-mer, L2-9-mer and Y1L2-9-mer, or Y1-10-mer) and used to stimulate autologous CD8+ T-cells in four rounds of stimulation over four weeks. Seven days after the fourth stimulation, CD8+ T-cells were challenged with the epitopes with which they had been stimulated or with the original epitopes. The original 9-mer induced specific CD8+ T cell response in only one out of eight donors (**Figure 3A**). Modification of this peptide at the position 1 with a tyrosine (Y1) did not improve immunogenicity, as the peptide elicited response in only one out of eight donors. Modification in the second (L2) or in the first and second (Y1L2) residues conferred high immunogenicity, as the epitopes containing such modifications elicited responses in three and two out of eight donors, respectively. The original 10-mer was immunogenic for half of the donors tested, which is in line with our previous experiments (**Figure 1D**). Substitution of the amino acid at position 1 with tyrosine improved immunogenicity of the 10-mer by 12.5%. While the immunogenicity of L2-9-mer and Y1L2-9-mer was improved, the Y1-9-mer was not more immunogenic than its original counterpart, despite its increased affinity towards the HLA-A*02:01. In addition to being more immunogenic than their original counterparts, the L2-9-mer and Y1L2-9-mer and Y1-10-mer were expected to be able to mobilize CD8+ T cell clones capable of recognizing the original G12V peptides. Modification in residue L2 not only rendered the peptide more immunogenic than its original 9-mer, but also rendered it capable of eliciting CD8+ T cells with ability to recognize the original 9-mer in five out of seven responding donors (**Figures 3B, C**). The 9-mer Y1L2 despite being more immunogenic than the original 9-mer, induced CD8+ T cells with capacity to recognize the original 9-mer in only one out of three donors (**Figure 3C**). Surprisingly, the Y1-10-mer despite being the most immunogenic of all G12V peptides, triggered T cells for the original 10-mer (O-10-mer) in only one out of six donors (**Figure 3C**). In addition to not being more immunogenic than its original counterpart, the 9-mer Y1 elicited CD8+ T cells that were not able to recognize the original 9-mer. Overall, the best RAS-derived peptides were the original 10-mer and the anchor-modified 9-mer L2 (**Figure 3D**).

### G12V-Specific CD8+ T Cells Recognize Peptide Presented by Pancreatic Cancer Cells

**Figure 4:**
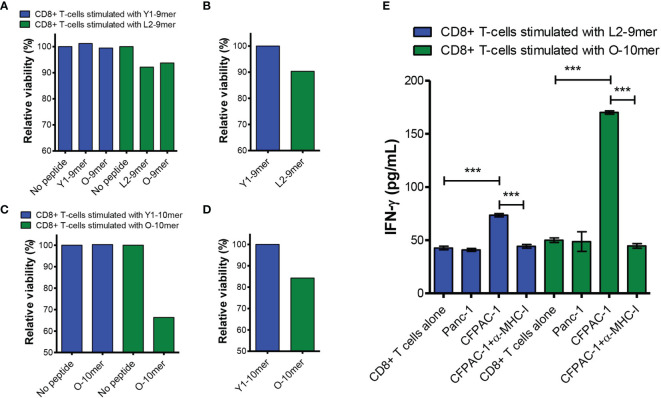
CD8+ T cells stimulated with L2-9-mer and O-10-mer G12V peptides recognize peptide presented by G12V-positive pancreatic cancer cells. CD8+ T cells isolated from an HLA-A*02:01-positive healthy donor blood were stimulated four times with autologous mature dendritic cells pulsed with Y1-9-mer or L2-9-mer peptides. Seven days after the last round of stimulation, CD8+ T cells were challenged with T2 cells pulsed with the same RAS mutants (anchor-modified or original), original or left without peptide **(A)** or with CFPAC-1 cells **(B)** and viability determined by staining the target cells with EthD-1 and measured by FACS. **(C, D)** CD8+ T-cells from another HLA-A*02:01-positive donor were stimulated with peptides Y1-10-mer or O-10-mer as in **(A, B)** and challenged with T2 cells pulsed with the same RAS peptides or left without peptide **(C)** or with CFPAC-1 cells **(D)**. **(E)** CD8+ T cells stimulated as in **(A–D)** with L2-9-mer or O-10-mer peptide were incubated overnight with G12V-negative/HLA-A*02:01-positive Panc-1 or G12V-positive/HLA-A*02:01-positive CFPAC-1 cells with or without blocking anti-MHC-I antibodies. Supernatants were collected and assayed for IFN-γ by ELISA. Data shown in **(E)** are from three independent repeats. The data were analyzed by one-way ANOVA; ***p< 0.001. Graph shows mean ± SEM.

We sought to investigate whether CD8+ T cells stimulated with L2-9mer and O-10-mer peptides have cytotoxic activity and can recognize tumor cells bearing the G12V mutation. To this end, CD8+ T-cells were generated from HLA-A*02:01-positive donors against the anchor-modified L2-9-mer and the O-10-mer G12V peptides. CTLs raised against L2-9-mer and O-10-mer peptides specifically lysed T2 cells pulsed with the appropriate peptide but did not lyse the unpulsed T2 target cells (**Figures 4A, C**). Most importantly, CTLs that were generated against the anchor-modified L2-9-mer peptide killed T2 cells pulsed with the original 9-mer peptide (**Figure 4A**). CTLs specific for the 9- and 10-mer G12V peptides were then tested for their ability to kill tumor cells that harbor the G12V mutation and express HLA-A*02:01. CTLs reactive to peptides L2-9-mer and 10-mer killed the human PDAC cell line CFPAC-1 that expresses both HLA-A*02:01 and G12V mutation (**Figures 4B, D**). CD8+ T-cells stimulated with Y1-9-mer or Y1-10-mer did not kill peptide-pulsed T2 or CFPAC-1 cells (**Figures 4B, D**), confirming the specificity of the CD8+ T-cells stimulated with L2-9-mer and O-10 towards cells presenting the original 9- and 10-mers. Moreover, incubation of L2-9-mer- and O-10-mer-stimulated CD8+ T cells with CFPAC-1 cells triggered IFN-γ secretion, which was decreased to baseline levels when anti-MHC-I antibodies were present, showing that the recognition of the tumor cells by the CD8+ T cells is HLA-restricted (**Figure 4E**). Baseline levels of IFN-γ were also observed when the CD8+ T cells were co-cultured with the G12V-negative and HLA-A*02:01-positive cell line Panc-1, indicating that the recognition is antigen-specific (**Figure 4E**). Overall, our data show that CD8+ T cells raised against the anchor-modified L2-9-mer and O-10-mer peptides can recognize and kill tumor cells expressing physiological levels of G12V-RAS peptide/MHC-I complex.

### Anchor-Modified G12V RAS Peptide Is Immunogenic *In Vivo*

**Figure 5:**
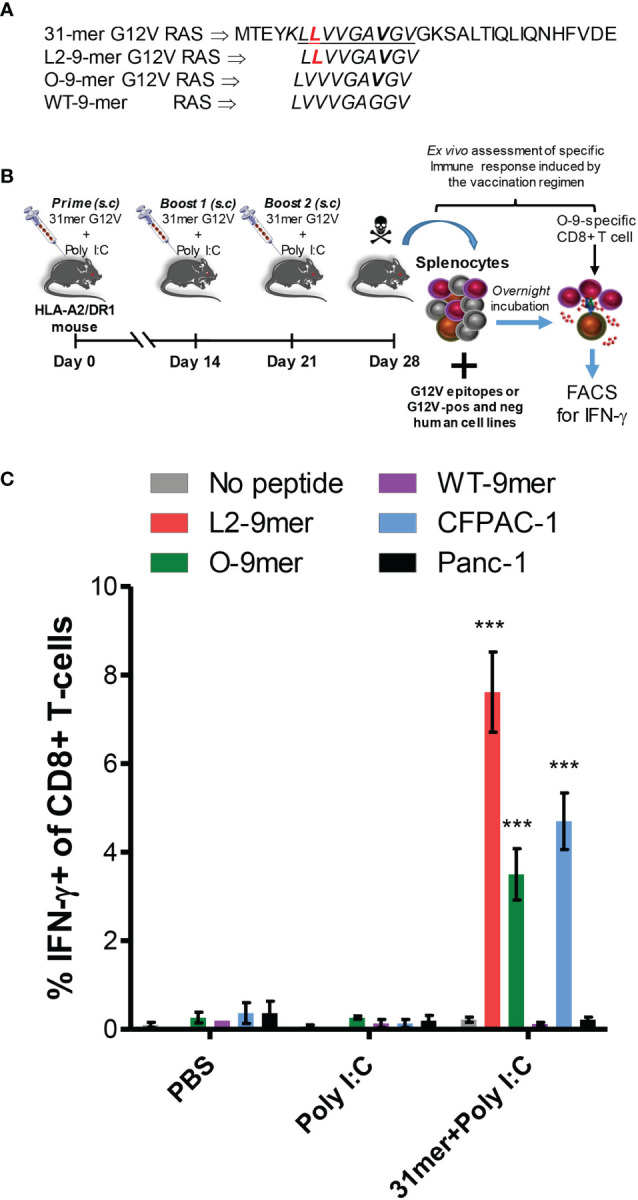
Immunogenicity of anchor-modified G12V RAS-mutant peptide *in vivo*. **(A)** Amino acid sequence of 31-mer G12V RAS long peptide, encompassing the L2-9-mer epitope; the L2-9-mer, O-9-mer and WT-9-mer epitopes. **(B)** Experimental set-up of vaccination of transgenic humanized HLA-A2/DR1 mice with the 31-mer G12V RAS peptide depicted in **(A)**. **(C)** Detection of intracellular IFN-γ in CD8+ T cells by FACS was used to assess *ex vivo* response of splenocytes of vaccinated mice following overnight incubation with the indicated minimal epitopes, the G12V-positive/HLA-A*02:01-positive or G12V-negative/HLA-A*02:01-positive Panc-1 cell lines. The graph shows mean ± SEM of experiments performed with 3 mice per group in PBS and Poly I:C groups (controls) and 5 mice in 31mer+Poly I:C group (experimental). The data were analyzed by one-way ANOVA; ***p< 0.001.

Next we sought to assess the immunogenicity of the anchor-modified L2-9-mer in an *in vivo* model. Peptide was synthesized as a 31-mer long peptide, containing the L2-9-mer minimal epitope (**Figure 5A**), and used together with poly I:C and Pan-DR peptide to immunize transgenic HLA-A2/DR1 mice (**Figure 5B**). Long peptide was used because we had observed previously that they are superior to short peptides in triggering peptide-specific CD8+ T cell responses (Manuscript in preparation). Robust response was observed following peptide vaccination, with about 8% of CD8+ T cells in the spleen of mice immunized with the 31-mer G12V peptide reacting to the minimal L2-9-mer peptide, half of which also reacted to the original 9-mer epitope. Importantly, the CD8+ T cells did not recognize the wild-type peptide. The CD8+ T cells recognized the HLA-A*02:01-positive/G12V-positive CFPAC-1 cells, but not the HLA-A*02:01-positive/G12V-negative Panc-1 cell line, showing the specificity of the immune response induced by the RAS peptide. Mice that received PBS or only poly I:C plus Pan-DR peptide exhibited negligible CD8+ T cell response against the peptides and cell lines (**Figure 5C**). In conclusion, these results show that vaccination with anchor-modified G12V peptide can elicit an efficient CD8+ T-cell-based immune response against the original 9-mer G12V epitope and should, therefore be considered in immunotherapeutic approaches for individuals with G12V-positive tumors.

## Discussion

The work described herein focused on determining the feasibility of targeting tumors harboring mutated RAS for immunotherapy. Using *in silico* algorithms, we identified neo-epitope candidates derived from the six most frequent RAS mutations in the context of HLA-A*02:01, HLA-A*03:01 and HLA-A*11:01 - alleles that together cover ~70% of the population worldwide. As the frequency of these six RAS mutations is around 20%, a 70% incidence of these three HLA-A alleles means that nearly 7 in 10 patients bearing RAS mutations could be eligible for protocols targeting these mutations. Unlike those HLA-A alleles, HLA-B and HLA-C alleles are not found in a large proportion of the population, with a few exceptions such as the HLA-B7 and HLA-C*08:02 – but even those alleles are only found in less than 10% of the population (29). For that reason we prioritized the HLA-A alleles with the highest frequency and that can bind mutant RAS-derived epitopes. The binding capacity of the predicted RAS neo-epitopes was further validated through cell-based binding assays followed by immunogenicity assays to determine their capacity to induce CD8+ T cells. We show that HLA-A*02:01- and HLA-A*03:01-binding peptides representing each of the six most common RAS mutations are immunogenic. There are reports documenting immunogenicity of G12V-derived peptides in the context of HLA-A*02:01 (17, 30, 31), but the capacity of the other mutations to trigger CD8+ T cells was largely unexplored. Here we show that the G12A and G12C mutations can generate HLA-A*02:01-binding 10-mer peptides that are as immunogenic as the G12V 10-mer peptide. This has enormous clinical relevance, considering that the G12C mutation is the most common in lung carcinoma (14), the leading cause of cancer death worldwide (32). The G12D mutation, which is the most frequent RAS alteration in the majority of cases (14) was also immunogenic in the context of HLA-A*02:01, albeit to a lesser extent than the G12A, G12C and G12V.

The two most frequent RAS alterations overall, the G12D and G12V were reported to elicit CD8+ T cell responses in the context of HLA-A*11:01 (19). In our study we found G12V to be immunogenic, but not G12D. This discrepancy could in part be due to the fact that in the reported study, they utilized an *in vivo* model with humanized mice expressing HLA-A*11:01, while we tested the immunogenicity of these peptides using an *in vitro* system. Besides, due to the lower frequency of HLA-A*11:01-positive donors in the UK compared to HLA-A*02:01 and HLA-A*03:01, we tested the immunogenicity of HLA-A*11:01-binding peptides in only three donors, which may reduce the likelihood of identifying less immunogenic peptides.

The HLA-A*03:01 and HLA-A*11:01 alleles belong to the same family and share similar binding capacity to many peptides (29). Our binding assays confirmed that the same RAS peptides could bind to both HLA-A*03:01 and HLA-A*11:01. Despite exhibiting high binding capacity to HLA-A*03:01, mutated RAS peptides have been largely neglected in the context of this HLA-A allele. T-cell assays revealed all six G12 RAS mutants as capable of triggering a CD8+ T cell response in HLA-A*03:01-positive subjects, which places them as viable targets for immunotherapeutic approaches.

Although RAS-derived epitopes were able to trigger peptide-specific CD8+ T cells in HLA-A*02:01- and HLA-A*03:01-positive donors, not all donors responded to all peptides, which could in part be explained by the limited T-cell repertoire for those MHC-I/peptide complexes in the general population. Alternatively, the lack of response could be due to the low to moderate affinity of such peptides for the HLA-A alleles. In view of these results, we sought to investigate if enhancing affinity could lead to increased immunogenicity of RAS epitopes with low or moderate affinity for HLA. Modifications in anchor residues have been attempted before with tumor-associated antigens (TAAs) (33–35). Vaccination of melanoma and lung cancer patients with modified TAAs-derived peptides induced peptide-specific immune responses, which correlated with improved clinical outcome (34). In our study we selected HLA-A*02:01-binding RAS peptides to test that approach, as HLA-A*02:01 is the most frequent HLA-I allele across most ethnic groups and its anchor motifs are very well-known (29). We made single and double alterations in positions 1 and 2 of G12 9-mers and a single alteration in position 1 of 10-mers. Placing a tyrosine in position 1 and/or a leucine in position 2 was reported to enhance affinity of TAA-derived peptides to HLA-A*02:01 without altering their specificity (33). The former feature was corroborated in our study, as these modifications were predicted to enhance affinity of the RAS peptides for HLA-A*02:01, which was subsequently confirmed by cell-binding assays. We next investigated whether the modifications could render the peptides more immunogenic without altering their antigenic specificity. The anchor-modified G12V peptides were able to trigger more vigorous CD8+ T-cell responses than their original counterparts, indicating that higher affinity translates into higher immunogenicity. However, while all modifications rendered vigorous CD8+ T cells against the anchor-modified peptides, most of them failed to induce CD8+ T cells capable of recognizing the original peptide. These results imply that anchor modifications can also alter specificity of the peptide, probably by altering its position inside the MHC-I groove, which could hide or expose regions in relation to the native peptide. This highlights the need for a thorough assessment of each anchor modification for their impact on antigen specificity.

In contrast to the modifications in position 1 and 1/2, the substitution of valine with leucine in position two of the G12V 9-mer fulfilled all requirements of a viable vaccine candidate, as it enhanced its binding affinity to HLA-A*02:01 and increased its immunogenicity, generating T-cells capable of recognizing the original mutated RAS epitope. Moreover, cytotoxic T cells raised against the L2-9-mer peptide lysed tumor cells positive for HLA-A*02:01 and bearing the G12V mutation. Our *in vitro* results were confirmed in an *in vivo* model in which transgenic humanized mice expressing HLA-A*02:01 were vaccinated with a long peptide containing the anchor-modified residue. The immunized mice generated a robust response against the L2-9-mer peptide with CD8+ T cells capable of recognizing the original 9-mer peptide and HLA-A*02:01-positive cancer cells bearing the G12V mutation. These data demonstrate that CD8+ T cells capable of reacting against this 9-mer mutant RAS peptide are present in the repertoire and can be recruited by a high affinity anchor-modified peptide. Additionally, these results strongly indicate that the MHC class I processing machinery of tumor cells is capable of generating and presenting the original G12V 9-mer on their cell surface for recognition by CTLs. Altogether, these results provide sound evidence that this anchor-modified peptide can be used in immunotherapy to treat G12V RAS-positive malignancies. To our knowledge, this is the first demonstration of this approach using a mutated antigen rather than a TAA to generate a CD8+ T cell-based response to cells presenting the original epitope.

To summarize, we showed that RAS peptides covering the six most common mutations were immunogenic in the context of HLA-A*02:01 and HLA-A*03:01. Moreover, modifications in anchor residues of all six RAS mutations enhanced affinity of the peptides to the HLA-A*02:01 molecule. Furthermore, immunogenicity of an HLA-A*02:01-binding 9-mer G12V peptide was greatly enhanced by substituting valine with leucine in position two, resulting in higher affinity of the peptide to the HLA and rendering it more immunogenic without losing specificity. Overall, the best HLA-A*02:01-binding G12V-derived peptides were the original 10-mer and the anchor-modified L2-9-mer. By using these two peptides, CD8+ T cell clones that recognize both 9-mer and 10-mer G12V RAS peptides can be engaged, providing the rationale for their use in a vaccine regimen or adoptive T-cell therapy targeting G12V-positive tumors. Future work is needed to assess the immunogenicity of anchor-modified peptides encompassing the other RAS mutations in the context of HLA-A*02:01, HLA-A*03:01 and HLA-A*11:01.

## Material and Methods

### Peptides

All peptides were custom-synthesized with a purity >98% by ProImmune Ltd (Oxford, United Kingdom) or GL Biochem Ltd (Shanghai, China). Lyophilized peptides were dissolved in DMSO (Pierce, Rockford, Illinois, USA) at 20 mg/mL and stored at −80°C.

### Cell-Based Binding Assays

Binding of HLA-A*02:01 and HLA-A*03:01 KRAS peptides was assessed in stabilization assays using TAP-deficient lymphoblastoid T2. For the assessment of HLA-A*11:01 peptides, a cell-based competitive binding assay with lymphoblastoid CJO-A cell line (European Collection of Authenticated Cell Cultures (ECACC), UK) was employed. T2 and T2 cells transfected with HLA-A*03:01 were kindly provided by Dr. Louise Boyle from University of Cambridge and by Professor Peter Cresswell from Yale University, respectively. All cell lines were grown and kept in culture in RPMI medium with 10% FCS at 37°C with 5% CO_2_. For the stabilization assays, the cells were washed and incubated in serum-free RPMI medium for 4 hours at 37°C with peptide concentrations ranging from 100 µM (in at least 8 serial dilution steps). The peptide-loaded stable MHC class I on the cell surface was measured by flow cytometry by staining the cells with fluorochrome-labelled monoclonal antibodies to HLA-A*02:01 (clone BB7.2; BD Biosciences), HLA-A*03:01 (clone GAP.A3; Fisher Scientific/eBioscience™). The mean fluorescence intensity (MFI) was taken as measure for the peptide stabilizing effect, thereby implicating peptide binding. EC50 values were calculated as the peptide concentration required for half-maximal MFI. For the cell-based competitive binding assay, lymphoblastoid CJO-A cells, homozygous for HLA-A*11:01 were incubated in serum-free RPMI medium for 4 hours at 37°C with peptide concentrations ranging from 100 µM (in at least 8 serial dilution steps) in the presence of 500 nM of FITC-labelled reference peptide KVFPK(FITC)ALINK (36). The relative binding of the unlabeled competitor peptides were expressed as inhibitory concentration (IC50), *i.e*., the concentration of competitor peptide required to inhibit 50% of binding of the FITC-labelled reference peptide. All measurements were performed with a FACSCalibur flow cytometer (BD Bioscience, Heidelberg, Germany). Data was analysed using WinMDi 2.9 (Purdue University, USA), and EC50/IC50 calculation with Graphpad Software Prism 5.04 for Windows (GraphPad Software, La Jolla, CA).

### Generation of DCs

Human DCs were differentiated from monocytic CD14+ precursors as described previously (37). Briefly, magnetic sorted CD14+ cells from PBMCs of healthy donors were cultured in 175 cm^2^ cell culture flasks in RPMI 1640 GlutaMax culture medium (Invitrogen, Carlsbad, CA, USA) with 10% heat-inactivated fetal bovine serum (FBS) (Invitrogen). For DC differentiation, on day 0 and 4 the cultures were supplemented with 50 ng/mL recombinant human GM-CSF and 50 ng/mL IL-4 (BioLegend). The cultures were maintained at 37°C in humidized atmosphere with 5% CO_2_. For DC maturation, on day 5 of culture, LPS (Sigma-Aldrich, Germany) at 100 ng/mL was added to the iDCs and the culture continued. The cells were harvested on day 7 and used in T cell stimulation assays.

### Immunogenicity T-Cell Assay and Generation of RAS-Specific CD8+ T-Cells

Immunogenicity of mutated RAS peptides was performed using HLA-matched healthy donors’ autologous PBMCs. Briefly, mature DCs were pulsed with individual mutated RAS epitopes at 25 μg/mL and co-cultured with autologous magnetic-sorted CD8+ T-cells (Miltenyi Biotec GmbH, Bergisch Gladbach, Germany) in RPMI with 5% human male AB serum, 10 units/mL penicillin–streptomycin, 2 mmol/L L-glutamine, 1% nonessential amino acid, IL-6 (1000 U/mL) and IL-12 (5 ng/mL). IL-2 (30 U/mL) and IL-15 (5 ng/mL) were added every 2 days. CD8+ T-cells underwent another 3 rounds of stimulation with peptide-pulsed CD14+ monocytes on day 7, 14 and 21. Seven days after the last stimulation, CD8+ T-cells were challenged by overnight incubation with peptide-loaded T2 cells or autologous monocytes. As a control, CD8+ T-cells were incubated with T2 cells or monocytes without peptide. Reactivity of the T cells was determined by intracellular IFN-γ staining measured by flow cytometry. In some experiments, cells were also stained with specific tetramers (BioLegend).

### Cytotoxicity Assay

For the cytotoxicity assays, the bulk of G12V RAS-stimulated CD8+ T-cells were incubated with peptide-loaded T2 cells or CFPAC-1 cell line. After overnight incubation, cells were stained with fluorochrome-conjugated anti-CD3 (BD), Calcein-AM and Ethidium Homodimer 1 (Invitrogen) to determine cell death. Target cells were gated on the CD3-negative population and cell death was determined by the percentage of cells negative for Calcein and/or positive for Ethidium Homodimer 1.

### ELISA for IFN-γ

The supernatants of co-cultures of tumor and CD8+ T-cells were collected after overnight incubation and stored at -80°C. IFN-γ levels from the co-cultures were measured by ELISA Max Standard kit (BioLegend) in 96-well microtiter plates according to the manufacturer’s instructions. Results are expressed as pg/mL.

### Flow Cytometry

For cell surface labelling, cell suspensions were incubated with antibodies or tetramers for 30 min at room temperature. For intracellular staining, cells were fixed and permeabilized using Leucoperm kit (Bio-Rad) in accordance with the manufacturer’s guidelines. Cell viability was analyzed by Ethidium homodimer 1 and calcein-AM double-staining using LIVE/DEAD^®^ Viability/Cytotoxicity Kit, for mammalian cells according to the manufacturer’s instructions (Invitrogen). Flow cytometry was done using a FACSCalibur (Becton Dickinson, Heidelberg, Germany) or BD LSRII (Becton Dickinson) flow cytometer. The data were processed with the CellQuest software (Becton Dickinson, Heidelberg, Germany) and analyzed with the WinMDi 2.9 (Purdue University, USA).

### Immunization of Transgenic Humanized HLA-A2/DR1 Mice With RAS Peptide

All mouse studies were carried out under the terms of the Home Office Project Licence PPL 70/6030 and subject to Queen Mary University of London ethical review, according to the guidelines for the welfare and use of animals in cancer research. Transgenic humanized HLA-A2/DR1 mice were obtained from Centre National de la Recherche Scientifique (CNRS), Transgénèse et Archivage d’Animaux Modèles (TAAM) (Orleans, France) and were bred and maintained in our animal facility at the Barts Cancer Institute. To test the immunogenicity of anchor-modified G12V RAS peptide, on days 0, 14 and 21, transgenic HLA-A2/DR1 mice were injected subcutaneously in the flank with 100 μg of the 31-mer G12V RAS_1-31_ peptide (MTEY*KL* ***L*** *VVGA* ***V*** *GV* GKSALTIQLIQNHFVDE) together with 25 μg of Poly I:C (*In vivo*gen) and 10 μg of Pan-DR peptide AKFVAAWTLKAAA. Control groups received either PBS or only poly I:C plus Pan-DR peptide. One week after the third injection the mice were culled, had their spleen harvested and used in *ex vivo* stimulation experiments.

### Splenocyte Preparation

Spleens were harvested from mice, combined with cell culture medium (RPMI medium, 10% FBS, 1% penicillin–streptomycin, and 1% sodium pyruvate), and single cell suspensions were prepared by mashing the spleen against a 70 μm cell strainer. Cells were resuspended in red blood cell lysis buffer (Sigma-Aldrich), washed in PBS, and the pellet was resuspended in cell culture medium.

### *Ex Vivo* Splenocyte Stimulation

Splenocytes (2 x 10^6^) were plated into each well of a U-bottom 96-well plate and restimulated with 10 μg/mL of the indicated peptides. Restimulated splenocytes were incubated overnight in the presence of brefeldin A (BFA; Sigma-Aldrich), and intracellular IFN-γ measured by flow cytometry.

### Statistical Analysis

Statistical analysis was carried out using the Graphpad Software Prism 5.01 or 7.03 for Windows (GraphPad Software, La Jolla, CA). The results were presented as mean ± standard error of the mean (SEM). Differences between groups were analyzed using one-way ANOVA test. Differences with a P<0.05 were considered significant.

### Ethics Statement for Use of Human PBMC

This study was reviewed and approved by the London-Westminster Research Ethics Committee (Protocol: 16/LO/1512) for access to healthy donor blood *via* the NHS blood and transplant service and all human materials used had written informed consent.

## Data Availability Statement

The original contributions presented in the study are included in the article/**Supplementary Material**. Further inquiries can be directed to the corresponding author.

## Ethics Statement

The animal study was reviewed and approved by the Queen Mary University of London ethical review. All mouse studies were carried out under the terms of the Home Office Project Licence PPL 70/6030 and subject to according to the guidelines for the welfare and use of animals in cancer research.

## Author Contributions

RBB and YW conceived of and designed the study. RBB, YL and PL developed the study methodology. RBB and PL conducted experiments and acquired data. RBB, PL and LSCD analyzed and interpreted the data (e.g., statistical analysis, biostatistics, and computational analysis). RBB, PL, LSCD, NL, and YW wrote, reviewed, and/or revised the manuscript. All authors contributed to the final version of the manuscript. YL and NL provided administrative, technical, or material support. YW supervised the study.

## Funding

This work was supported by grants from the Pancreatic Cancer Research Fund (PCRF), grant no to RBB, PL and YW Cancer Research UK (CRUK, grant number C15051/A27224), to SL, LSCD and YW.

## Conflict of Interest

The authors declare that the research was conducted in the absence of any commercial or financial relationships that could be construed as a potential conflict of interest.

## Publisher’s Note

All claims expressed in this article are solely those of the authors and do not necessarily represent those of their affiliated organizations, or those of the publisher, the editors and the reviewers. Any product that may be evaluated in this article, or claim that may be made by its manufacturer, is not guaranteed or endorsed by the publisher.

## References

[1] Hamdy S Haddadi A Hung RW Lavasanifar A. Targeting Dendritic Cells With Nano-Particulate PLGA Cancer Vaccine Formulations. Adv Drug Deliv Rev (2011) 63(10-11):943–55. doi: 10.1016/j.addr.2011.05.021 21679733

[2] Sheng WY Huang L. Cancer Immunotherapy and Nanomedicine. Pharm Res (2011) 28(2):200–14. doi: 10.1007/s11095-010-0258-8 20821040

[3] Matsuo Y Takeyama H Guha S. Cytokine Network: New Targeted Therapy for Pancreatic Cancer. Curr Pharm Des (2012) 18(17):2416–9. doi: 10.2174/13816128112092416 22372505

[4] Gubin MM Zhang X Schuster H Caron E Ward JP Noguchi T. Checkpoint Blockade Cancer Immunotherapy Targets Tumour-Specific Mutant Antigens. Nature (2014) 515(7528):577–81. doi: 10.1038/nature13988 PMC4279952 25428507

[5] Lollini PL Cavallo F Nanni P Quaglino E. The Promise of Preventive Cancer Vaccines. Vaccines (Basel) (2015) 3(2):467–89. doi: 10.3390/vaccines3020467 PMC4494347 26343198

[6] Melero I Gaudernack G Gerritsen W Huber C Parmiani G Scholl S. Therapeutic Vaccines for Cancer: An Overview of Clinical Trials. Nat Rev Clin Oncol (2014) 11:509. doi: 10.1038/nrclinonc.2014.111 25001465

[7] Li L Goedegebuure SP Gillanders WE. Preclinical and Clinical Development of Neoantigen Vaccines. Ann Oncol (2017) 28(suppl_12):xii11–7. doi: 10.1093/annonc/mdx681 PMC5834106 29253113

[8] Rogers MF Gaunt TR Campbell C. CScape-Somatic: Distinguishing Driver and Passenger Point Mutations in the Cancer Genome. Bioinformatics (2020) 36:btaa242. doi: 10.1093/bioinformatics/btab654 PMC7320610 32282885

[9] Prior IA Hood FE Hartley JL. The Frequency of Ras Mutations in Cancer. Cancer Res (2020) 80:2969–74. doi: 10.1158/0008-5472.CAN-19-3682 PMC7367715 32209560

[10] Quinlan MP Settleman J. Isoform-Specific Ras Functions in Development and Cancer. Future Oncol (2009) 5:105–16. doi: 10.2217/14796694.5.1.105 19243303

[11] Balachandran VP Luksza M Zhao JN Makarov V Moral JA Remark R. Identification of Unique Neoantigen Qualities in Long-Term Survivors of Pancreatic Cancer. Nature (2017) 551(7681):512–6. doi: 10.1038/nature24462 PMC6145146 29132146

[12] Witkiewicz AK McMillan EA Balaji U Baek G Lin WC Mansour J. Whole-Exome Sequencing of Pancreatic Cancer Defines Genetic Diversity and Therapeutic Targets. Nat Commun (2015) 6:6744. doi: 10.1038/ncomms7744 25855536 PMC4403382

[13] Waddell N Pajic M Patch AM Chang DK Kassahn KS Bailey P. Whole Genomes Redefine the Mutational Landscape of Pancreatic Cancer. Nature (2015) 518(7540):495–501. doi: 10.1038/nature14169 25719666 PMC4523082

[14] Prior IA Lewis PD Mattos C. A Comprehensive Survey of Ras Mutations in Cancer. Cancer Res (2012) 72(10):2457–67. doi: 10.1158/0008-5472.CAN-11-2612 PMC3354961 22589270

[15] Jonckheere N Vasseur R Van Seuningen I. The Cornerstone K-RAS Mutation in Pancreatic Adenocarcinoma: From Cell Signaling Network, Target Genes, Biological Processes to Therapeutic Targeting. Crit Rev Oncol Hematol (2017) 111:7–19. doi: 10.1016/j.critrevonc.2017.01.002 28259298

[16] Janes MR Zhang J Li LS Hansen R Peters U Guo X. Targeting KRAS Mutant Cancers With a Covalent G12C-Specific Inhibitor. Cell (2018) 172(3):578–89. doi: 10.1016/j.cell.2018.01.006 29373830

[17] Kubuschok B Neumann F Breit R Sester M Schormann C Wagner C. Naturally Occurring T-Cell Response Against Mutated P21 Ras Oncoprotein in Pancreatic Cancer. Clin Cancer Res (2006) 12(4):1365–72. doi: 10.1158/1078-0432.CCR-05-1672 16489095

[18] Tran E Robbins PF Lu YC Prickett TD Gartner JJ Jia L. T-Cell Transfer Therapy Targeting Mutant KRAS in Cancer. N Engl J Med (2016) 375(23):2255–62. doi: 10.1056/NEJMoa1609279 PMC5178827 27959684

[19] Wang QJ Yu Z Griffith K Hanada K Restifo NP Yang JC. Identification of T-Cell Receptors Targeting KRAS-Mutated Human Tumors. Cancer Immunol Res (2016) 4(3):204–14. doi: 10.1158/2326-6066.CIR-15-0188 PMC4775432 26701267

[20] Gjertsen MK Buanes T Rosseland AR Bakka A Gladhaug I Søreide O. Intradermal Ras Peptide Vaccination With Granulocyte-Macrophage Colony-Stimulating Factor as Adjuvant: Clinical and Immunological Responses in Patients With Pancreatic Adenocarcinoma. Int J Cancer (2001) 93(3):441–50. doi: 10.1002/ijc.1205 11291084

[21] Weden S Klemp M Gladhaug IP Moller M Eriksen JA Gaudernack G. Long-Term Follow-Up of Patients With Resected Pancreatic Cancer Following Vaccination Against Mutant K-Ras. Int J Cancer (2011) 128(5):1120–8. doi: 10.1002/ijc.25449 20473937

[22] Eriksen JA Gladhaug IP Rosseland A Risberg Handeland K Buanes T. An Observational Clinical Study With RAS Peptide Vaccine TG01 Evaluating Immune Response, Safety and Overall Survival in Patients With non-Resectable Pancreatic Cancer. Ann Oncol (2017) 28(suppl_5):mdx376.018–mdx376.018. doi: 10.1093/annonc/mdx361

[23] Parker KC Bednarek MA Hull LK Utz U Cunningham B Zweerink HJ. Sequence Motifs Important for Peptide Binding to the Human MHC Class I Molecule, HLA-A2. J Immunol (1992) 149:3580–7. 1331239

[24] Rammensee HG Friede T Stevanoviic S. MHC Ligands and Peptide Motifs: First Listing. Immunogenetics (1995) 41:178–228. doi: 10.1007/BF00172063 7890324

[25] Schultze JL Michalak S Seamon MJ Dranoff G Jung K Daley J. CD40-Activated Human B Cells: An Alternative Source of Highly Efficient Antigen Presenting Cells to Generate Autologous Antigen-Specific T Cells for Adoptive Immunotherapy. J Clin Investig (1997) 100:2757–2765. doi: 10.1172/JCI119822 9389740 PMC508480

[26] Tourdot S Oukka M Manuguerra JC Magafa V Vergnon I Riche N. Chimeric Peptides: A New Approach to Enhancing the Immunogenicity of Peptides With Low MHC Class I Affinity: Application in Antiviral Vaccination. J Immunol (1997) 159:2391–8. 9278330

[27] Pardoll DM. Inducing Autoimmune Disease to Treat Cancer. Proc Natl Acad Sci USA (1999) 96:5340–2. doi: 10.1073/pnas.96.10.5340 PMC33572 10318881

[28] Andreatta M Nielsen M. Gapped Sequence Alignment Using Artificial Neural Networks: Application to the MHC Class I System. Bioinformatics (2016) 32(4):511–7. doi: 10.1093/bioinformatics/btv639 PMC6402319 26515819

[29] Sette A Sidney J. Nine Major HLA Class I Supertypes Account for the Vast Preponderance of HLA-A and -B Polymorphism. Immunogenetics (1999) 50:201–12. doi: 10.1007/s002510050594 10602880

[30] Bergmann-Leitner ES Kantor JA Shupert WL Schlom J Abrams SI. Identification of a Human CD8+ T Lymphocyte Neo-Epitope Created by a Ras Codon 12 Mutation Which Is Restricted by the HLA-A2 Allele. Cell Immunol (1998) 187(2):103–16. doi: 10.1006/cimm.1998.1325 9732698

[31] Escobar P Yu Z Terskikh A Holmes N Corradin G Mach JP. Induction in Transgenic Mice of HLA-A2.1-Restricted Cytotoxic T Cells Specific for a Peptide Sequence From a Mutated P21ras Protein. Clin Exp Immunol (1999) 116(2):214–9. doi: 10.1046/j.1365-2249.1999.00873.x PMC1905280 10337009

[32] Torre LA Siegel RL Jemal A. Lung Cancer Statistics. Adv Exp Med Biol (2016) 893:1–19. doi: 10.1007/978-3-319-24223-1_1 26667336

[33] Tourdot S Scardino A A Saloustrou E Gross DA Pascolo S Cordopatis P. A General Strategy to Enhance Immunogenicity of Low-Affinity HLA-A2.1-Associated Peptides: Implication in the Identification of Cryptic Tumor Epitopes. Eur J Immunol (2000) 30(12):3411–21. doi: 10.1002/1521-4141(2000012)30:12<3411::AID-IMMU3411>3.0.CO;2-R 11093159

[34] Kotsakis A Papadimitraki E Vetsika EK Aggouraki D Dermitzaki EK Hatzidaki D. A Phase II Trial Evaluating the Clinical and Immunologic Response of HLA-A2+ Non Small Cell Lung Cancer Patients Vaccinated With an Htert Cryptic Peptide. Lung Cancer (2014) 86(1):59–66. doi: 10.1016/j.lungcan.2014.07.018 25130084

[35] Gallou C Rougeot A Graff-Dubois S Kosmatopoulos K Menez-Jamet J. A General Strategy to Optimize Immunogenicity of HLA-B*0702 Restricted Cryptic Peptides From Tumor Associated Antigens: Design of Universal Neo-Antigen Like Tumor Vaccines for HLA-B*0702 Positive Patients. Oncotarget (2016) 7(37):59417–28. doi: 10.18632/oncotarget.11086 PMC5312321 27506946

[36] Kessler JH Mommaas B Mutis T Huijbers I Vissers D Benckhuijsen WE. Competition-Based Cellular Peptide Binding Assays for 13 Prevalent HLA Class I Alleles Using Fluorescein-Labeled Synthetic Peptides. Hum Immunol (2003) 64(2):245–55. doi: 10.1016/S0198-8859(02)00787-5 12559627

[37] Baleeiro RB Wiesmüller KH Dähne L Lademann J Barbuto JA Walden P. Direct Activation of Human Dendritic Cells by Particle-Bound But Not Soluble MHC Class II Ligand. PloS One (2013) 8(5):e63039. doi: 10.1371/journal.pone.0063039 23658796 PMC3642081

