# Supplementary material for: Optimized Anchor-Modified Peptides Targeting Mutated RAS Are Promising Candidates for Immunotherapy
Source: Front Immunol. 2022 May 26;13:902709. doi: 10.3389/fimmu.2022.902709 (PMC9204602; doi:10.3389/fimmu.2022.902709)
Supplement: Supplementary file 1 [file DataSheet_1.pdf]

Table S1 - Binding affinity of mutant RAS peptides was predicted by NetMHC 4.0 program and affinity in nM and %Rank are shown.

HLA	Peptide ID	Sequence	Aff (nM) NetMHC	Rank (%) NetMHC
HLA-A*02:01	G12A (9mer)	LVVVGA**A**GV	1519.99	5
	G12A (10mer)	KLVVVGA**A**GV	237.8	1.8
	G12C (9mer)	LVVVGA**C**GV	2088.83	6
	G12C (10mer)	KLVVVGA**C**GV	373.6	2.5
	G12D (9mer)	LVVVGA**D**GV	4317.54	9
	G12D (10mer)	KLVVVGA**D**GV	498	3
	G12R (9mer)	LVVVGA**R**GV	8610.82	14
	G12R (10mer)	KLVVVGA**R**GV	506.9	3
	G12S (9mer)	LVVVGA**S**GV	3297.93	7.5
	G12S (10mer)	KLVVVGA**S**GV	390.7	2.5
	G12V (9mer)	LVVVGA**V**GV	1710.31	5.5
	G12V (10mer)	KLVVVGA**V**GV	300.2	2
HLA-A*03:01	G12A (9mer)	VVGA**A**GVGK	363.7	1.1
	G12A (10mer)	VVVGA**A**GVGK	492.9	1.3
	G12C (9mer)	VVGA**C**GVGK	221.4	0.8
	G12C (10mer)	VVVGA**C**GVGK	375.5	1.1
	G12D (9mer)	VVGA**D**GVGK	1172.08	2.5
	G12D (10mer)	VVVGA**D**GVGK	938.8	1.9
	G12R (9mer)	VVGA**R**GVGK	140.1	0.6
	G12R (10mer)	VVVGA**R**GVGK	246	0.9
	G12S (9mer)	VVGA**S**GVGK	216.3	0.8
	G12S (10mer)	VVVGA**S**GVGK	355.3	1.1
	G12V (9mer)	VVGA**V**GVGK	202.6	0.8
	G12V (10mer)	VVVGA**V**GVGK	342	1.1
HLA-A*11:01	G12A (9mer)	VVGA**A**GVGK	147.7	0.9
	G12A (10mer)	VVVGA**A**GVGK	243.1	1.2
	G12C (9mer)	VVGA**C**GVGK	135	0.8
	G12C (10mer)	VVVGA**C**GVGK	278.8	1.3
	G12D (9mer)	VVGA**D**GVGK	368.19	1.5
	G12D (10mer)	VVVGA**D**GVGK	430	1.7
	G12R (9mer)	VVGA**R**GVGK	163.1	1
	G12R (10mer)	VVVGA**R**GVGK	308.5	1.4
	G12S (9mer)	VVGA**S**GVGK	114.4	0.8
	G12S (10mer)	VVVGA**S**GVGK	213.1	1.1
	G12V (9mer)	VVGA**V**GVGK	65.5	0.5
	G12V (10mer)	VVVGA**V**GVGK	137.3	0.9

Table S2 - Binding affinity of mutant RAS peptides was predicted by NetMHC 4.0 program and affinity in nM and %Rank are shown. The actual binding capacity of the peptides to HLA-A*02:01, HLA-A*03:01 and HLA-A*11:01 was subsequently assessed in cell binding assays and EC50 (for HLA-A*02:01 and HLA-A*03:01 peptides) or IC50 (for HLA-A*11:01 peptides) values in μM for each peptide are shown. Peptides highlighted in blue were used as positive controls for the binding assays.

HLA	Peptide ID	Sequence	Aff (nM) NetMHC	Rank (%) NetMHC	*EC/IC50 (μM) Binding assay
HLA-A*02:01	G12A (10mer)	KLVVVGA**A**GV	237.8	1.8	50.22
	G12C (10mer)	KLVVVGA**C**GV	373.6	2.5	95.93
	G12D (10mer)	KLVVVGA**D**GV	498	3	97.12
	G12R (10mer)	KLVVVGA**R**GV	506.9	3	151.2
	G12S (10mer)	KLVVVGA**S**GV	390.7	2.5	71.96
	G12V (10mer)	KLVVVGA**V**GV	300.2	2	35.81
	Flu-MP 58-66	***GILGFVFTL***	***15.71***	***0.2***	***0.8797***
HLA-A*03:01	G12A (9mer)	VVGA**A**GVGK	363.7	1.1	277.2
	G12A (10mer)	VVVGA**A**GVGK	492.9	1.3	94.03
	G12C (9mer)	VVGA**C**GVGK	221.4	0.8	828.3
	G12C (10mer)	VVVGA**C**GVGK	375.5	1.1	142.6
	G12D (10mer)	VVVGA**D**GVGK	938.8	1.9	998.8
	G12R (9mer)	VVGA**R**GVGK	140.1	0.6	695.2
	G12R (10mer)	VVVGA**R**GVGK	246	0.9	69.73
	G12S (9mer)	VVGA**S**GVGK	216.3	0.8	174.2
	G12S (10mer)	VVVGA**S**GVGK	355.3	1.1	78.04
	G12V (9mer)	VVGA**V**GVGK	202.6	0.8	110.1
	G12V (10mer)	VVVGA**V**GVGK	342	1.1	63.91
	Flu-NP 265-273	***ILRGSVAHK***	***19.94***	***0.08***	***6.33***
HLA-A*11:01	G12A (9mer)	VVGA**A**GVGK	147.7	0.9	11.23
	G12A (10mer)	VVVGA**A**GVGK	243.1	1.2	7.03
	G12C (9mer)	VVGA**C**GVGK	135	0.8	29.08
	G12C (10mer)	VVVGA**C**GVGK	278.8	1.3	15.63
	G12D (10mer)	VVVGA**D**GVGK	430	1.7	15.03
	G12R (9mer)	VVGA**R**GVGK	163.1	1	156.6
	G12R (10mer)	VVVGA**R**GVGK	308.5	1.4	46.14
	G12S (9mer)	VVGA**S**GVGK	114.4	0.8	12.71
	G12S (10mer)	VVVGA**S**GVGK	213.1	1.1	10.23
	G12V (9mer)	VVGA**V**GVGK	65.5	0.5	5.071
	G12V (10mer)	VVVGA**V**GVGK	137.3	0.9	6.954
	Flu-MP 13-21	***SIIPSGPLK***	***8***	***0.01***	***1.441***
